# Caspase-1 Mediated Cleavage of BMP Type I Receptor Drives BMP2-Induced Differentiation of Bone Marrow Mesenchymal Stem Cells into Adipocytes

**DOI:** 10.3390/app152413105

**Published:** 2025-12-12

**Authors:** Kelechi Chukwuocha, Venu Pandit, Daniel Halloran, Anja Nohe

**Affiliations:** 1 Department of Biological Sciences, University of Delaware, Newark, DE 19716, USA; 2 Children’s Hospital of Philadelphia, Philadelphia, PA 19104, USA

**Keywords:** stem cell differentiation, osteoporosis, BMP2, BMPRIa, adipocytes, osteoblasts, PPARγ, Caspase-1

## Abstract

Bone Morphogenetic Protein-2 (BMP2) is a growth factor that maintains bone homeostasis through the BMP receptor type Ia (BMPRIa) and type II (BMPRII). BMP2 promotes osteogenesis by inducing the differentiation of bone marrow mesenchymal stem cells (BMSCs) into osteoblasts; however, it can also trigger BMSC differentiation into adipocytes. BMP2’s osteo-inductive ability has made it a potential treatment for osteoporosis, yet its dual role in BMSC differentiation complicates its efficacy. High BMP2 levels cause BMPRIa cleavage, but the downstream effects and the mechanisms governing BMP2-induced osteogenesis or adipogenesis are unresolved. Here, we identify Caspase-1 as a key mediator of BMPRIa cleavage and its downstream effects on adipogenesis. We used primary BMSCs from C57BL/6 mice, stimulated with varying BMP2 concentrations, to explore BMP2-induced BMPRIa cleavage and its impact on PPARγ—a key regulator of adipogenesis. Western blotting and immunostaining using antibodies against BMPRIa and PPARγ uncovered BMPRIa cleavage and revealed the nuclear translocation of the cleaved segment, colocalizing with PPARγ. Caspase-1 inhibition significantly reduced BMPRIa cleavage and PPARγ expression, highlighting its pivotal role in adipogenic differentiation. Understanding the molecular mechanisms of BMP2-induced adipogenesis and Caspase-1 inhibition could improve BMP2 therapeutic efficacy for osteoporosis by promoting osteogenesis over adipogenesis.

## 1. Introduction

Bone marrow mesenchymal stem cells (BMSCs) are a heterogeneous population of multipotent cells residing in the bone marrow [1]. These cells play a crucial role in the maintenance and repair of musculoskeletal tissues due to their ability to differentiate into multiple cell types, including osteoblasts, chondrocytes, and adipocytes. BMSCs are integral to skeletal health, influencing bone formation, remodeling, and supporting hematopoiesis [2-4]. Osteoblasts, which are derived from BMSCs, are responsible for synthesizing and mineralizing the bone matrix [5,6]. The differentiation of BMSCs into osteoblasts is a tightly regulated process involving various signaling pathways, with bone morphogenetic proteins (BMPs), particularly BMP2, playing a central role.

In addition to osteoblast differentiation, BMSCs can also differentiate into adipocytes, the fat-storing cells found in marrow adipose tissue (MAT). This process of adipogenic differentiation is influenced by several factors, including the transcription factor Peroxisome Proliferator-Activated Receptor Gamma (PPARγ), which is critical in regulating adipogenesis [7,8], as well as adipokines such as adiponectin (Adipoq), which further promote adipocyte function and differentiation. Conversely, osteogenesis is favored by increased activity of osteogenic transcription factors such as Runt-related transcription factor 2 (Runx2) and Osterix (Osx), which promote commitment toward the osteoblastic lineage [9]. The balance between osteogenesis and adipogenesis in BMSCs is primarily regulated by PPARγ activity, with increased PPARγ favoring adipocyte formation. Thus, modulating PPARγ activity is crucial in determining whether BMSCs will contribute to bone formation or fat accumulation within the bone marrow [8]. An imbalance favoring adipogenesis can negatively impact bone density and strength, leading to conditions such as osteoporosis (OP) [10-12].

Osteoporosis is a severe bone disorder that affects over 10 million Americans, with 80% of those diagnosed being women [13-15]. OP is characterized by decreased bone mineral density (BMD) and results from an imbalance between bone formation and resorption [16,17]. This disorder is costly, diminishes quality of life, increases mortality, and remains incurable. It is also age-related, as there is a preferential increase in the differentiation of BMSCs into adipocytes rather than osteoblasts during aging [18,19].

Current treatment options primarily focus on stimulating bone formation (anabolic therapies) or slowing down bone degradation (anti-resorptive therapies). Anti-resorptive agents, such as bisphosphonates (e.g., alendronate and risedronate), inhibit osteoclast-mediated bone resorption, thereby increasing bone mineral density. Another commonly used anti-resorptive treatment is denosumab, a monoclonal antibody that targets RANKL (Receptor Activator of Nuclear factor Kappa-B Ligand), preventing osteoclast formation and function [20]. On the anabolic side, romosozumab is a recently approved monoclonal antibody that inhibits sclerostin, a negative regulator of bone formation. Romosozumab not only promotes osteoblast activity and new bone formation but also reduces bone resorption, offering a dual mechanism of action. Despite their efficacy, these therapies are often associated with adverse effects, including osteonecrosis of the jaw (ONJ), atypical femoral fractures, osteolysis, and hematoma formation [21,22]. Thus, there is an urgent need for safer treatment strategies that effectively enhance bone formation while minimizing the risk of adverse side effects.

One potential therapeutic target for OP is BMP signaling, particularly BMP2, which plays a critical role in regulating BMSC differentiation [23,24]. BMPs are members of the transforming growth factor-β (TGF-β) superfamily and regulate various cellular processes including osteogenic differentiation, chondrogenesis, cell proliferation, and apoptosis [23,25-27]. Specifically, BMP2, first identified by Dr. Marshall Urist in 1965, is a crucial growth factor for BMSC differentiation [26]. BMP2 has gained attention for its osteoinductive properties, making it a candidate for osteoporosis therapy [13,28]. In 2002, the FDA approved recombinant human BMP2 (rhBMP2) for specific surgical procedures, such as anterior lumbar interbody fusion (ALIF) as well as cranioplasties, and bone regeneration after maxillofacial injuries [29]. BMP2 exerts its effects by binding to specific receptors on the cell surface, including Bone Morphogenetic Protein Receptor Type IA (BMPRIa) [30-32]. Upon BMP2 binding, BMPRIa undergoes phosphorylation and forms complexes with other receptors, activating downstream signaling pathways that regulate gene expression and cellular differentiation [33]. This highlights the importance of BMP receptors in the BMP2 signaling process [34].

BMP receptors are primarily localized on the plasma membrane of cells and can signal through both canonical Smad-dependent and Smad-independent pathways. In the Smad-independent pathway, BMPRIa activates mitogen-activated protein kinase (MAPK) and phosphatidylinositol 3-kinase (PI3K) pathways, promoting cellular survival and proliferation [35]. In Smad-dependent signaling, BMP2 binds to BMPRIa, which is typically localized in caveolae enriched with caveolin-1 alpha isoforms or within clathrin-coated pits (CCPs). BMPRII is then recruited to the membrane or is already localized to the membrane domains with BMPRIa as pre-formed complexes [36]. This dynamic shuttling of receptors between different caveolar isoforms within the plasma membrane is crucial for enhancing Smad signaling, emphasizing the importance of receptor localization in the signaling process [37-39]. BMPRII then phosphorylates BMPRIa, releasing protein kinase CK2, and subsequently BMPRIa phosphorylates downstream proteins, including SMAD1, 5, and 8 [5,40]. The phosphorylated SMADs recruit SMAD4, forming a complex that translocates to the nucleus to act as transcription factors that activate target genes [41-43].

Despite the initial promise of BMP2 as a therapeutic agent for osteoporosis, subsequent studies have raised concerns about its safety and efficacy [44,45]. Complications associated with rhBMP2 therapy include increased bone resorption, nerve injuries, and altered bone quality resulting from increased adipocyte formation and decreased trabecular spacing [44,46]. These issues prompted the FDA to issue a black box warning in 2008, emphasizing the need for caution with rhBMP2 administration [46].

To address these complexities, mouse models devoid of confounding variables offer clearer insights into BMP2’s therapeutic potential in osteoporosis. Using mouse models, we aim to unravel the pathophysiology of BMP2-induced differentiation of BMSCs into adipocytes, which poses a challenge to the efficacy of BMP2, and to optimize therapeutic strategies for conditions like osteoporosis. Previous studies using the immortalized murine myoblast cell line (C2C12) have shown that high BMP2 concentrations lead to the proteolytic cleavage of BMPRIa [42,47], possibly mediated by Caspase-1. Caspase-1 is a key component of the inflammasome, mediating the maturation and secretion of proinflammatory cytokines such as interleukin-1β (IL-1β) and interleukin-18 (IL-18), which are essential in various physiological and pathological processes [48,49]. Caspase-1’s role in BMP2-mediated processes like bone formation and adipocyte differentiation suggests its involvement in modulating the balance between osteogenesis and adipogenesis [49,50]. Furthermore, at a high BMP2 concentration (specifically 200 nM), C2C12 cells differentiate into adipocytes, while at 40 nM BMP2, they form more osteoblasts [47].

A critical question that remains unclear is what protease mediates BMPRIa cleavage and how this event influences the switch between osteogenic and adipogenic differentiation. Although the downstream effects of this proteolytic cleavage and the involvement of Caspase-1, along with the effects of these BMP2 concentrations on PPARγ, which is the key regulator of BMSC differentiation into adipocytes, have not been confirmed, this study seeks to elucidate these aspects further. Accordingly, this work investigates the roles of BMPRIa and PPARγ in regulating the osteogenesis-adipogenesis balance in BMSCs, with a particular focus on identifying the protease responsible for BMPRIa cleavage and the downstream effects of this cleavage.

Understanding the mechanisms by which BMP2 induces BMSC differentiation into adipocytes is crucial for developing therapies to prevent or treat osteoporosis, where excessive bone marrow adipogenesis contributes to bone weakening. This study aims to elucidate the roles of BMPRIa, PPARγ, and other molecular players in regulating the balance between osteogenesis and adipogenesis in BMSCs.

## 2. Materials and Methods

### 2.1. Immortalized Murine Myoblast Cell Line (C2C12)

C2C12 cells were acquired from The American Type Culture Collection (ATCC; Manassas, VA, USA). This cell line originates from murine myoblast progenitors and was subsequently immortalized. C2C12 cells predominantly differentiate into myocytes under basal conditions but can be directed toward osteogenic or adipogenic lineages upon BMP2 stimulation. In this study, they were utilized specifically for antibody validation and confirmation of BMPRIa cleavage.

### 2.2. C2C12 BMPRIa Knockout Cell Line

A C2C12 BMPRIa knockout cell line was generated using ribonucleoprotein (RNP, spCas9 + gRNA) delivery via 4D nucleofection by the ChristianaCare Gene Editing Institute in Newark, DE, USA. This process resulted in the creation of a bulk population of C2C12 cells harboring targeted mutations in exon 2 of the BMPRIa gene. Specifically, two clones (clones 4 and 6) were derived from this population using the same gRNAs (gRNA 3: CACUGGUAUGAAAUCAGACU and gRNA 5: UGUUAUUAAUAGCAUCAUCU). A 94-base pair deletion spanning the cut sites was induced, resulting in a frameshift mutation and premature stop codon. Analysis conducted 48 h post-transfection confirmed the presence of the frameshift mutation across the entire cell population through Sanger sequencing. The cell population was subsequently single-cell sorted into 96-well plates using the Namocell instrument (Namocell, Mountain View, CA, USA). Upon confluency in the 96-well plates, each clone was expanded, and Sanger sequencing was performed to identify clones with frameshift knockouts (KO). Clones exhibiting the desired frameshift KO profile were further expanded and frozen. Confirmation of the knockout was obtained through additional Sanger sequencing and the use of deconvolution of Complex DNA Repair (DECODR) by Dr. Byung-Chun Yoo and London McGill of the Gene Editing Institute.

### 2.3. Mice Subjects

Following approval from the University of Delaware Institutional Animal Care and Use Committee (IACUC, AUP #1194), female C57BL/6 (B6) mice were sourced from Charles River Laboratories (Horsham, PA, USA) via the National Institute of Aging (NIA). The mice were 6 months old upon arrival. Subsequently, they were housed five mice per cage in the Life Science Research Facility at the University of Delaware (Newark, DE, USA) for one week to acclimate.

### 2.4. Euthanasia and Isolation of Bone Marrow Stem Cells from B6 Mice

The mice were euthanized using CO_2_ followed by cervical dislocation. To isolate the BMSCs, the femurs were initially dissected at the distal and proximal femoral heads. Subsequently, the bones were flushed with alpha minimum essential media (αMEM; Caisson Labs, Smithfield, UT, USA) and filtered using a 70 μm cell strainer (Stellar Scientific, Baltimore, MD, USA) into 50 mL conical tubes (Cole-Parmer, Vernon Hills, IL, USA). The cells were then centrifuged at 1500 rpm for 5 min at 4 °C and resuspended in 5 mL of αMEM.

### 2.5. C2C12 Cell Culture

C2C12 cells, both the wild-type and BMPRIa knockout (KO) cells, were cultured to approximately 70–80% confluency in T75 flasks using Dulbecco’s Modified Eagle’s Medium (DMEM; HyClone, Pittsburgh, PA, USA) supplemented with 10% Fetal Bovine Serum (FBS; Gemini Bioproducts, West Sacramento, CA, USA), 1% antibiotic/antimycotic (anti/anti; Gemini Bioproducts, West Sacramento, CA, USA), and 1% penicillin/streptomycin (pen/strep; Fisher Scientific, Pittsburg, PA, USA). Upon reaching confluency, cells were then seeded into either 12- or 24-well plates (Nest Scientific, Woodbridge Township, NJ, USA) at a density of 1 × 10^6^ cells/mL for 12-well plates or 1 × 10^5^ cells/mL for 24-well plates.

### 2.6. Primary Cell Culture

After isolating BMSCs from the femurs of B6 mice, the cells were cultured in 12-well plates at a density of 1 × 10^6^ cells/mL in αMEM supplemented with 10% FBS, 1% penicillin/streptomycin (pen/strep), and 1% antibiotic/antimycotic solution for 7 days. For the immunostaining experiments, cells were plated at 1 × 10^6^ cells/mL in 12-well plates on 18 mm diameter cover slips (Catalog #CS-R18-100, Amscope, Irvine, CA, USA) and grown for a total of 10–14 days before staining. Similarly, for Western blot analysis, BMSCs were seeded in 12-well plates at a density of 1 × 10^6^ cells/mL and cultured in αMEM containing 10% FBS, 1% pen/strep, and 1% antibiotic/antimycotic for 10–14 days before lysate collection.

### 2.7. Immunostaining of Primary Cells

BMSCs were isolated from 10 B6 mice, each 6 months old. The cells were plated at a density of 1 × 10^6^ cells/mL and allowed to attach for two days. Subsequently, media changes were performed on day 3 and day 6 of culture. On day 6, the cells were treated with or without 12 μM of Caspase-1 inhibitor I (Ac-YVAD-CHO; Catalog# sc-358878A, Santa Cruz Biotechnology, Inc., Dallas, TX, USA) for 24 h. Ac-YVAD-CHO was dissolved in sterile water; therefore, a vehicle-only control was not required. Following this treatment, the cells were stimulated with either 40 nM BMP2, 200 nM BMP2, or left unstimulated (control group) for an additional 24 h. BMP2 was reconstituted in sterile water; thus, no vehicle control was necessary for BMP2 treatments. The media in the well-plates was then aspirated, and cells were washed with ice-cold 1× PBS. Subsequently, the cells were fixed with 4.4% paraformaldehyde (PFA; pH 7.2; Sigma Aldrich, St. Louis, MO, USA) for 15 min at room temperature. After fixation, the cells were washed three times with ice-cold 1× PBS and permeabilized for 10 min with 0.1% Saponin (Sigma Aldrich, St. Louis, MO, USA) dissolved in sterile water. Following permeabilization, cells were blocked with 3% bovine serum albumin (BSA; Fisher Scientific, Pittsburgh, PA, USA) containing 0.1% Saponin in 1× PBS for one hour on ice. Subsequently, all cells except for the secondary control were incubated with primary mouse monoclonal BMPRIa antibody (Catalog # sc-293175; Santa Cruz Biotechnology, Inc., Dallas, TX, USA) and rabbit polyclonal PPARγ antibody (Catalog # 16643-1-AP; Proteintech Group, Inc., Rosemont, IL, USA) at a 1:100 dilution in 1× PBS containing 0.1% Saponin and 3% BSA for one hour on ice. Following primary antibody incubation, cells were washed three times with 1× PBS and incubated with secondary antibodies (donkey-anti-mouse Alexa Fluor^™^ 594 and chicken-anti-rabbit Alexa Fluor^™^ 488, both from Invitrogen, Eugene, OR, USA) at a 1:500 dilution in 1× PBS containing 0.1% Saponin and 3% BSA. After additional washing with 1× PBS, cells were treated with Hoechst 33342 (Catalog #AR0039, Bolster Bio, Pleasanton, CA, USA) for 7.5 min. Finally, coverslips were mounted using Airvol mounting medium and allowed to dry before imaging with the Zeiss LSM880 confocal microscope with Airy scan (Wolf Hall, University of Delaware, Newark, DE, USA) using a 63x objective. This experiment was performed in triplicate, acquiring 10 images for each experimental condition, and all data were normalized to the secondary control.

### 2.8. Immunofluorescence Quantification

For semi-quantitative analysis of immunostaining fluorescence, Fiji (ImageJ2; Version 2.16.0/1.54p; National Institutes of Health, Bethesda, MD, USA) was employed. Background measurements from the micrographs were initially obtained and subtracted from the fluorescent intensity values. The resulting corrected intensities were then averaged for each experimental group. Subsequently, the data were normalized to the secondary control of the respective experiments to account for any variations in staining or imaging conditions.

### 2.9. Lysate Collection for Western Blotting

Cells were cultured to confluency in T25 flasks at a density of 2.5 × 10^6^ cells. The cells were then treated with or without 12 μM of Caspase-1 inhibitor I (Ac-YVAD-CHO; Catalog# sc-358878A, Santa Cruz Biotechnology, Inc., Dallas, TX, USA) for 24 h. Following this treatment, the cells were stimulated with either 40 nM BMP2, 200 nM BMP2, or left unstimulated (control group) for an additional 24 h. After stimulation, the media was removed, and the cells were washed three times with 1× PBS. Subsequently, the cells were lysed for 10 min with RIPA lysis buffer (0.44 g NaCl, 500 μL Triton X-100, 0.05 g sodium deoxycholate, 12.5 mL Tris 0.5 M, 0.05 g SDS, 37 mL sterile water) supplemented with Halt^™^ Protease Inhibitor Cocktail (100×; Catalog #78425; Thermo Fisher Scientific Inc., Waltham, MA, USA). The lysates were sonicated at 40% amplitude using a digital sonifier (Digital Sonifier Model 250; BRANSON Ultrasonics Corporation, Danbury, Connecticut, USA). The lysates were then centrifuged at 12,700 rpm for 10 min using an Eppendorf Refrigerated Centrifuge (Eppendorf 5702R; Marshall Scientific Inc., Hampton, NH, USA).

### 2.10. Western Blotting

Protein concentrations of the lysates were determined by BCA assay and normalized prior to boiling for 10 min at 95 °C. Equal volumes (20 μL) corresponding to equal amounts of protein were then loaded into a 10% SDS-PAGE gel. Gel electrophoresis was carried out at 120 V for 1 h and 30 min and the proteins were transferred to a PVDF (Immobilon, Darmstadt, Germany) membrane for 1 h and 10 min at 15 V using the semi-dry transfer machine (BioRad, Hercules, CA, USA). The membrane was blocked with 5% BSA diluted in 1× Tris-Buffered Saline-Tween (TBST) for 1 h on ice. The blot was incubated overnight at 4 °C with a 1:1000 dilution of BMPRIa primary antibody (same as above). On the following day, the blot was washed three times with 1× TBST and incubated with a 1:5000 dilution of secondary antibody (same as the above) for 1 h at room temperature. The blots were washed three more times with 1× TBST and treated with SuperSignalTM West Pico Plus Chemiluminescent Substrate (Thermo Scientific, Rockford, IL, USA) for 5 min. All protein bands were detected with the Invitrogen iBright1500 machine (Thermo Fisher Scientific, Waltham, MA, USA).

### 2.11. Oil Red O Staining Protocol

Primary BMSCs were isolated from 6-month-old mice and cultured to 90% confluence in 24-well plates and serum-starved overnight before treatment. Caspase-1 inhibitor I (Ac-YVAD-CHO) was used to inhibit Caspase-1 activity in the designated groups prior to BMP2 stimulation. The following day, cells were washed with cold PBS (pH 7.4), fixed using 4% (*w/v*) paraformaldehyde for 10 min, and washed again with cold PBS (pH 7.4) to remove excess fixative. Oil Red O stain was prepared by creating a stock solution consisting of 0.35 g of Oil Red O powder dissolved in 100 mL isopropanol. On the day of staining, 6 mL of the stock solution was mixed with 4 mL dH2O to make the working solution, which was set aside for 20 min and then filtered through a 0.22 μm filter. The working solution was applied to each well for 15 min, followed by a 1 min wash with dH2O to avoid washing off lipid droplets. Plates were allowed to dry. The area covered by lipid droplets (identified by the red/dark stain) was analyzed by taking random high magnification images of each well using a light microscope. Data were quantified using ImageJ software, where images were converted to 8-bit, and the threshold was set to the positive control. The same threshold was used for all treatments in an individual experiment.

### 2.12. Statistical Analysis

All data were subjected to single-factor analysis of variance (ANOVA). Subsequently, Tukey–Kramer HSD, Student’s *t*-test, and/or Fisher’s exact statistical tests were applied where appropriate. Error bars on bar graphs represent the standard deviation (SD). Statistical significance was defined as *p* < 0.05, denoted by an asterisk (*) indicating significant differences between groups. Significance levels are indicated as follows: *p* < 0.05 = (*), *p* < 0.01 = (**), *p* < 0.001 = (***), *p* < 0.0001 = (****); *n.s.* = not significant. Experiments were repeated three or more times, and all data were normalized to the respective control group within each experiment.

## 3. Results

### 3.1. BMP2-Induced Cleavage of BMPRIa in C2C12 Cells

To determine whether BMP2 stimulation induces proteolytic cleavage of the BMPRIa receptor, C2C12 cells were treated with increasing concentrations of BMP2 (0, 40, and 200 nM), followed by SDS–PAGE and Western blotting using an anti-BMPRIa antibody. The selected BMP2 concentrations were based on previous findings by [47], who reported that low BMP2 levels (40 nM) promote osteogenic differentiation, whereas higher concentrations (200 nM) favor adipogenic signaling.

**Figure 1.**
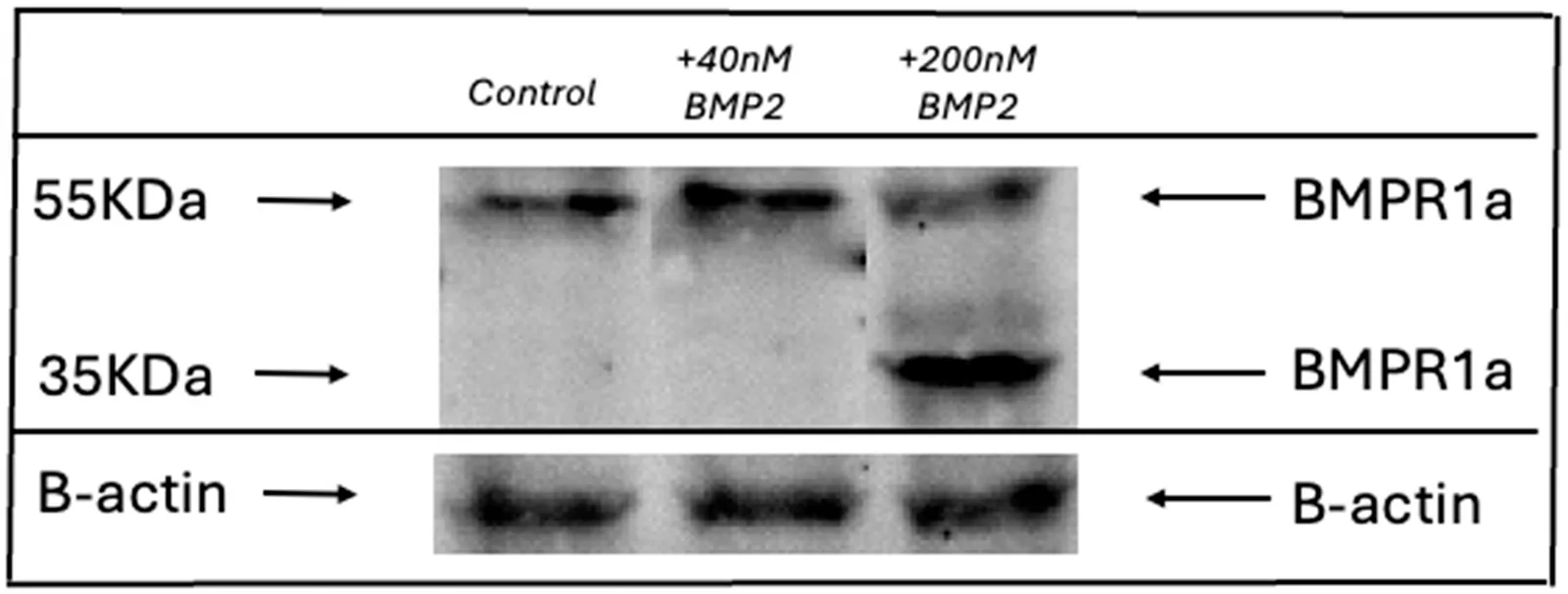
BMP2-induced cleavage of BMPRIa in C2C12 cells. Western blot analysis showing BMPRIa expression in C2C12 cells stimulated with BMP2 (0, 40, 200 nM). Full-length BMPRIa (~55 kDa) was detected under all conditions, while a distinct ~35 kDa cleavage fragment appeared prominently following 200 nM BMP2 stimulation, indicating BMP2-induced receptor cleavage. β-actin (42 kDa) served as a loading control.

As shown in Figure 1, two immunoreactive bands were detected: a major band corresponding to the full-length BMPRIa (~55 kDa) and a lower-molecular-weight band at ~35 kDa, which became markedly more prominent following 200 nM BMP2 stimulation. β-actin served as a loading control to verify equal protein loading across samples. The appearance of the 35 kDa fragment at high BMP2 concentration indicates ligand-induced cleavage of BMPRIa, consistent with concentration-dependent receptor processing.

These findings demonstrate that high BMP2 levels (200 nM) promote BMPRIa cleavage in C2C12 cells, suggesting that BMP2 concentration influences receptor stability and signaling outcomes. This observation prompted further investigation into whether similar cleavage events occur in primary bone marrow mesenchymal stem cells (BMSCs), which more closely represent in vivo cellular responses.

### 3.2. BMP2 Stimulation at 200 nM Leads to an Increase in PPARγ Expression in Primary BMSCs

**Figure 2.**
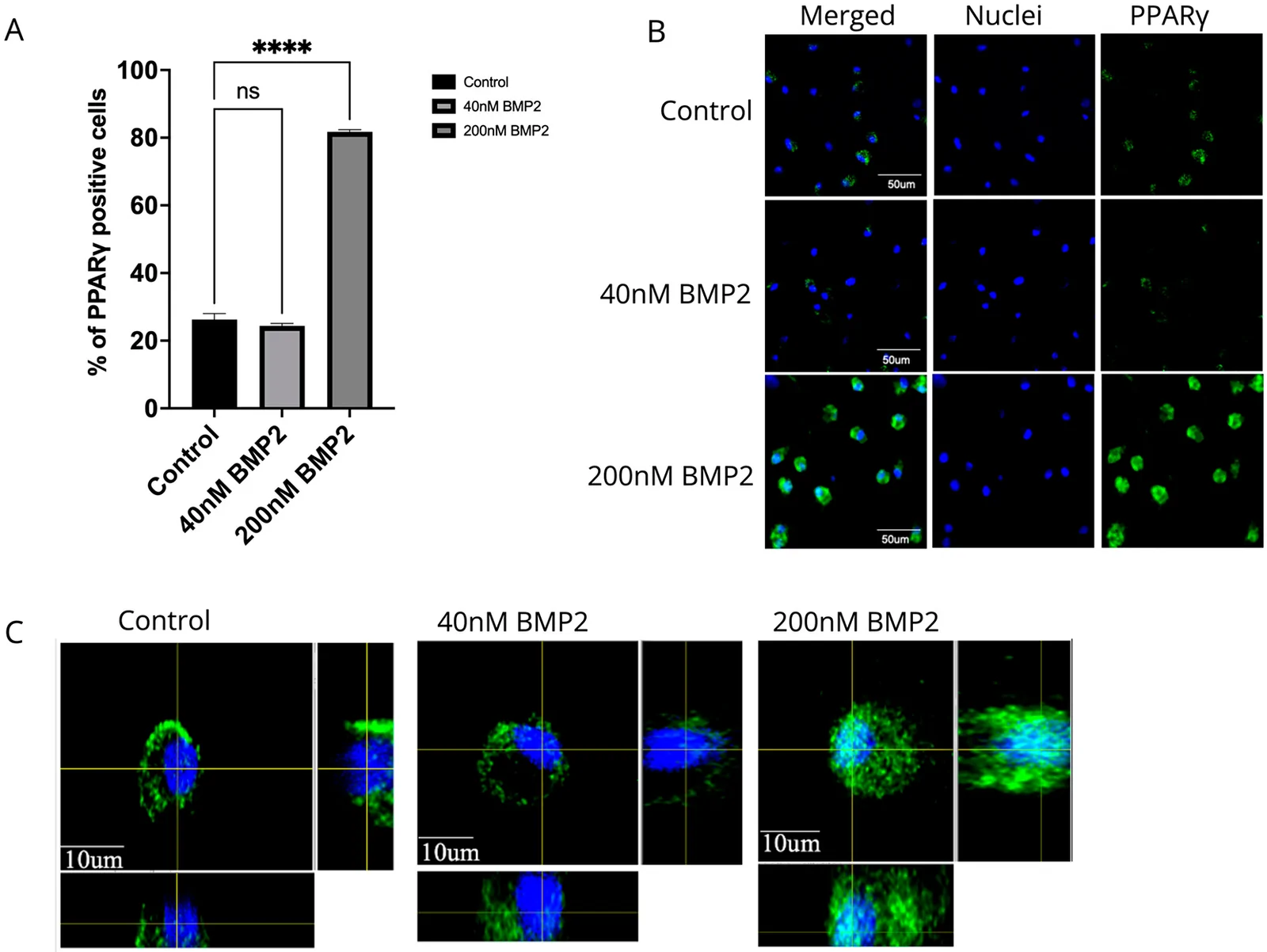
PPARγ expression in primary BMSCs stimulated with BMP2. (**A**) Quantification of PPARγ-positive cells following BMP2 treatment (0, 40, and 200 nM), **** = *p* < 0.0001 and *n.s.* = not significant. (**B**) Representative immunofluorescence images showing PPARγ (green) and nuclei (Hoechst, blue). Scale bars = 50 μm. (**C**) Higher-resolution z-stack orthogonal views confirming nuclear localization of PPARγ. Scale bars = 10 μm.

To investigate the effects of BMP2 stimulation on PPARγ expression in BMSCs, primary BMSCs were isolated from the femurs of 6-month-old C57BL/6 mice, cultured, and stained with antibodies specific to PPARγ. Treatment with a high dose of BMP2 (200 nM) resulted in a marked increase in the number of PPARγ-positive cells compared to the control (0 nM) and 40 nM BMP2 groups, both of which showed minimal PPARγ expression (Figure 2A,B). Using confocal microscopy, we obtained high-resolution images of the cells and quantified the proportion of individual nuclei expressing PPARγ. As PPARγ is a master regulator of adipogenesis, PPARγ-positive cells were considered preadipocytes, whereas PPARγ-negative cells likely represented pre-osteoblasts. Random fields were imaged, and approximately 140 cells were analyzed per group across three independent experiments (*n* = 3), totaling ~420 cells (*p* < 0.05).

As shown in Figure 2A, approximately 82% of cells in the 200 nM BMP2 group were PPARγ-positive, compared to less than ~25% in the control and 40 nM BMP2 groups, both of which had a greater proportion of PPARγ-negative cells. Furthermore, PPARγ expression was observed to be more nuclear in the 200 nM BMP2 stimulated group when compared to a more cytoplasmic expression observed in the 0 and 40 nM BMP2 treatment groups as shown in Figure 2B,C.

These findings indicate that high BMP2 concentration (200 nM) significantly increases the expression of PPARγ in primary BMSCs, suggesting a potential shift towards adipogenesis. Conversely, low BMP2 concentration (40 nM) maintains minimal PPARγ expression. This consolidates the findings by [47] that 200 nM BMP2 promotes adipogenesis, whereas lower BMP2 concentrations favor osteogenic differentiation. Together, these results highlight a dose-dependent regulation of BMSC fate by BMP2.

### 3.3. BMP2 Stimulation at 200 nM Concentration Leads to the Nuclear Accumulation of BMPRIa in BMSCs

As stated previously, BMP2 exerts its effects through the BMPRIa receptor, which is typically localized on the plasma membrane of cells. Previous research by [42] found that BMP2 stimulation leads to the cleavage of the BMPRIa receptor in C2C12 cells. Here, we investigated whether BMP2 stimulation alters BMPRIa localization in primary BMSCs. BMSCs were isolated from C57BL/6 mice and treated with BMP2 at 0 nM, 40 nM, or 200 nM. Cells were stained with antibodies specific for BMPRIa and PPARγ, and high-resolution confocal images were obtained for analysis.

**Figure 3.**
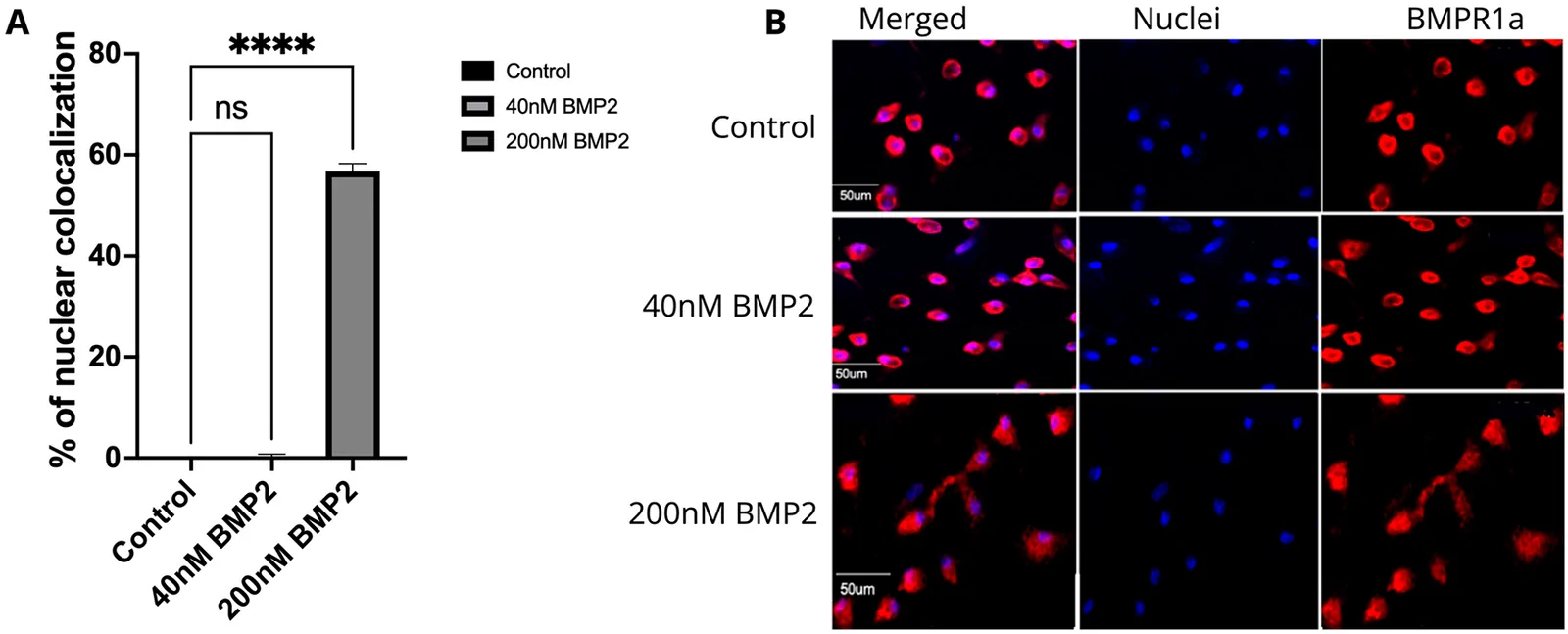

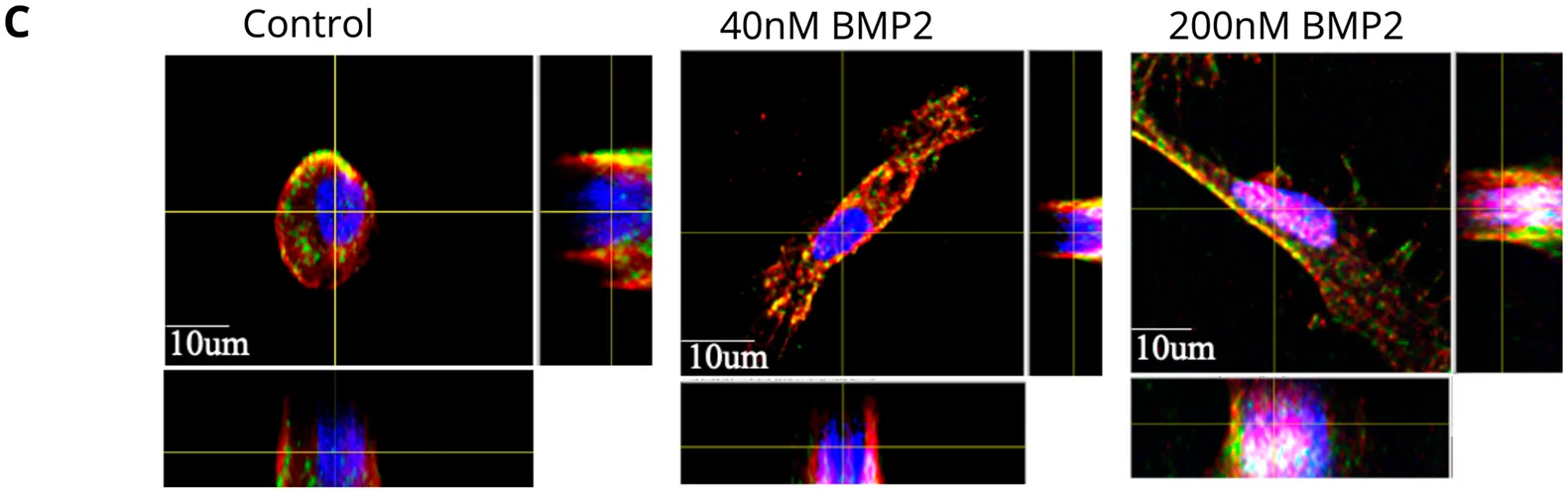
BMP2 stimulation induces nuclear accumulation of BMPRIa in primary BMSCs. (**A**) Quantification of BMPRIa nuclear localization (% of nuclear colocalization) in BMSCs treated with BMP2 (0, 40, or 200 nM). Data represent mean ± SEM from three independent experiments (*n* = 3; **** = *p* < 0.0001 and *n.s.* = not significant.). (**B**) Representative immunofluorescence images showing BMPRIa (red) and nuclei (Hoechst, blue). Cells were treated with BMP2 at the indicated concentrations. Scale bars = 50 μm. (**C**) High-resolution z-stack orthogonal images showing increased nuclear accumulation of BMPRIa (red) in the 200 nM BMP2 group compared to the 0 and 40 nM groups. Scale bars = 10 μm.

Quantitative analysis revealed a significant increase in the percentage of cells showing nuclear localization of BMPRIa at 200 nM BMP2 compared to the 0 nM and 40 nM groups (Figure 3A). At control and 40 nM BMP2, BMPRIa was primarily detected at the cell membrane and in the cytoplasm, consistent with its canonical signaling role (Figure 3B). In contrast, at 200 nM BMP2, BMPRIa exhibited a prominent redistribution into the nuclear region, resulting in nuclear colocalization of BMPRIa and PPARγ (Figure 3C).

This data demonstrates that BMP2 concentration influences the localization of BMPRIa in primary BMSCs. At lower concentrations (0 and 40 nM), BMPRIa remains primarily at the cell membrane and cytoplasmic region. However, at a higher concentration (200 nM), BMPRIa accumulates in the nuclear region, suggesting a potential cleavage of the receptor and a role for nuclear translocation in BMP2-induced signaling pathways.

### 3.4. Identification of Caspase-1 as a BMPRIa Protease

To further investigate the potential cleavage of BMPRIa and identify the specific protease involved, we performed an in silico peptide cleavage analysis using the ExPASy PeptideCutter tool (SIB Swiss Institute of Bioinformatics, Lausanne, Switzerland). The results indicated that Caspase-1 and prolyl endopeptidase were the two enzymes predicted to cleave BMPRIa at its C-terminal region. Caspase-1 was predicted to produce two fragments, whereas prolyl endopeptidase would generate three [51].

**Figure 4.**
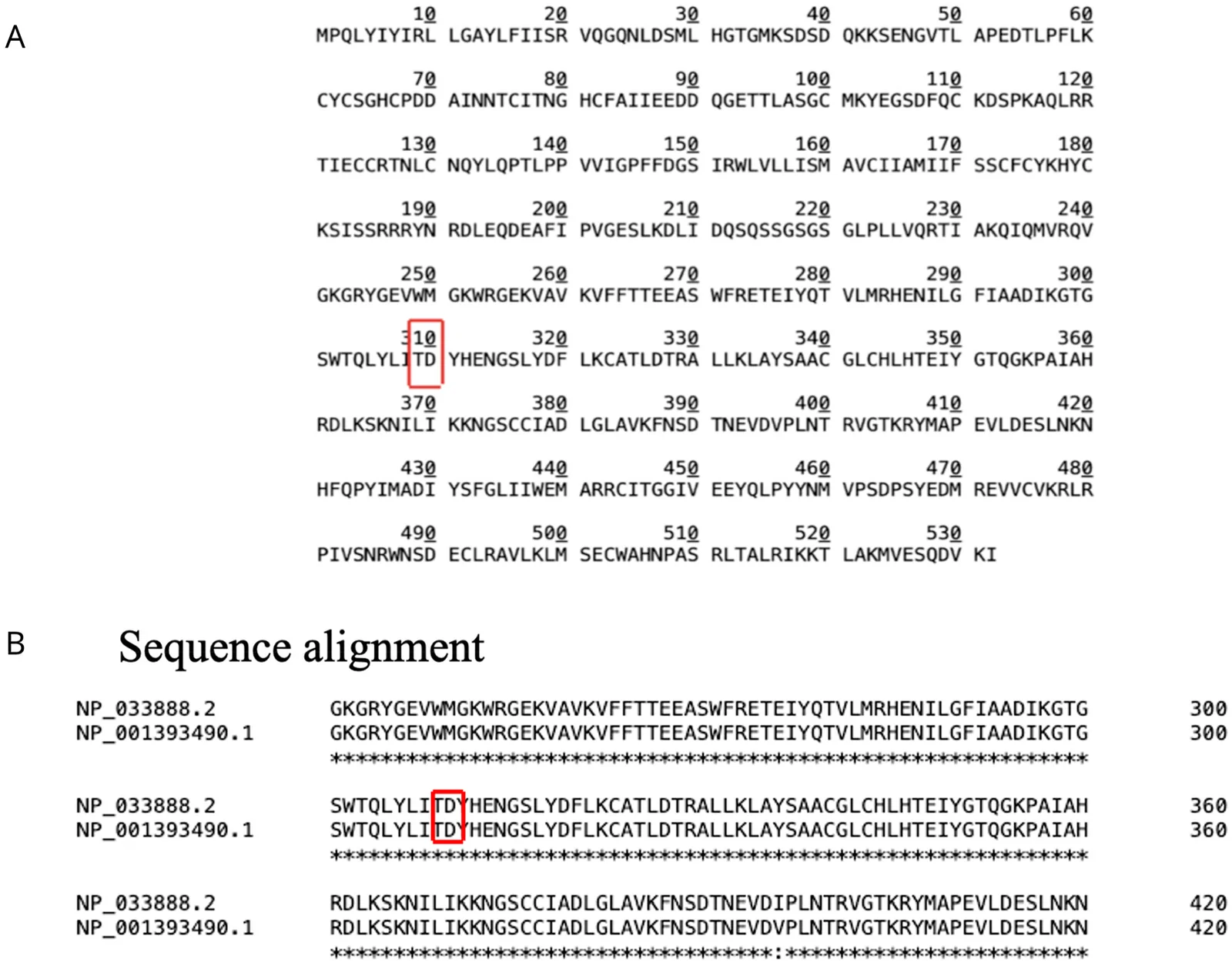
Predicted Caspase-1 cleavage site on murine BMPRIa. (**A**) Full-length mouse BMPRIa amino acid sequence (532 aa; NCBI Accession NP_033888.2) analyzed using the ExPASy PeptideCutter tool. A predicted Caspase-1 cleavage site was identified at Asp^310^ (boxed), located near the receptor’s C-terminal cytoplasmic domain. (**B**) Pairwise sequence alignment of mouse BMPRIa (NP_033888.2) and human BMPRIa (NP_001393490.1) showing complete conservation of the Caspase-1 cleavage motif, supporting the evolutionary conservation and translational relevance of Caspase-1–mediated BMPRIa processing.

However, previous studies by [52] reported that prolyl endopeptidase preferentially cleaves short peptide substrates (<30 amino acids) due to the structural constraints imposed by its β-propeller domain. Given that BMPRIa has a 532 amino acid sequence (Figure 4), it is unlikely to be a substrate for prolyl endopeptidase. Furthermore, our Western blot data demonstrated that BMP2 treatment leads to two distinct BMPRIa fragments (~55 kDa and ~35 kDa), consistent with Caspase-1–mediated cleavage rather than prolyl endopeptidase activity. Importantly, Caspase-1 was predicted to cleave BMPRIa at the 310 amino acid residue (Figure 4), located near the C-terminal cytoplasmic region of the receptor. This cleavage site corresponds to the approximate molecular weight of the 35 kDa fragment detected in our immunoblot analysis. Such cleavage likely releases a C-terminal fragment capable of nuclear translocation, consistent with the nuclear accumulation of BMPRIa observed in Figure 3. The BMPRIa amino acid sequence analyzed in Figure 4 corresponds to the murine BMPRIa (encoded by the Bmpr1a gene, NCBI Accession NP_033888.2), consistent with the use of primary mouse BMSCs in this study. Sequence alignment with human BMPRIa (NP_001393490.1) revealed over 97% identity, with complete conservation surrounding the Caspase-1 cleavage site at Asp^310^.

This high degree of homology suggests that Caspase-1–mediated cleavage of BMPRIa is likely conserved across species, supporting the translational relevance of these findings to human BMP2 signaling.

Together, these results identify Caspase-1 as a likely BMPRIa protease, providing a mechanistic link between BMP2-induced receptor cleavage and subsequent nuclear localization. This supports the hypothesis that Caspase-1–mediated cleavage of BMPRIa contributes to the regulation of BMP2 signaling and adipogenic differentiation in primary BMSCs.

### 3.5. BMP2 Induces BMPRIa Cleavage, and Inhibition of Caspase-1 Abolished the Cleavage in Primary BMSCs

To ascertain the cleavage of BMPRIa in primary cells, and the involvement of Caspase-1, we isolated BMSCs from the femurs of 6-month-old C57BL/6 mice. The cells were divided into two major groups: Caspase-1 inhibited and non-inhibited groups. Each group was subsequently split into 0, 40, and 200 nM BMP2 treatment groups as in previous experiments. The Caspase-1 inhibitor Ac-YVAD-CHO was used to inhibit the activity of Caspase-1 prior to BMP2 stimulation in the Caspase-1 inhibited group.

**Figure 5.**
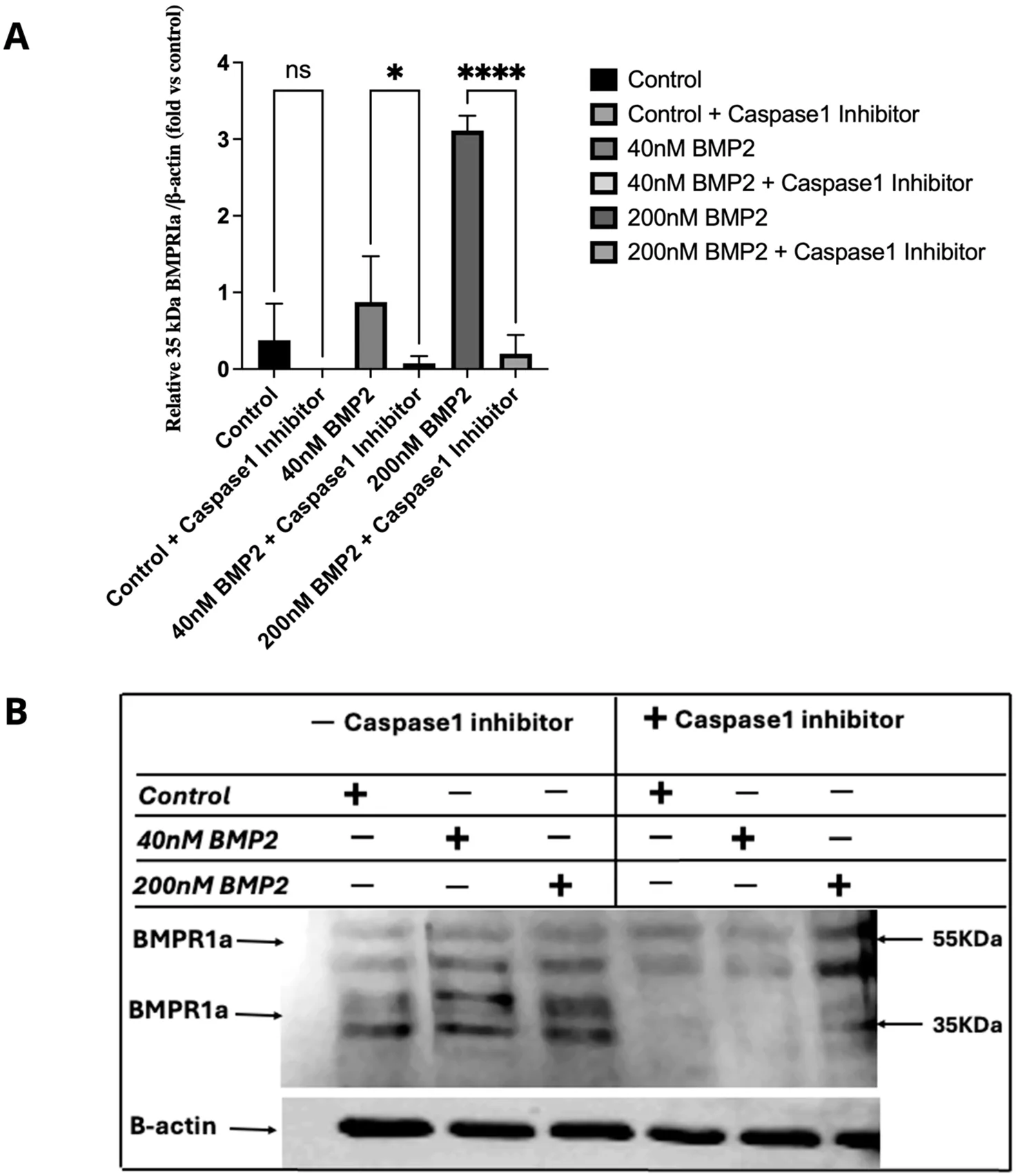
BMP2-induced BMPRIa cleavage is attenuated by Caspase-1 inhibition in primary BMSCs. (**A**) Western blot analysis showing BMPRIa cleavage in primary BMSCs treated with BMP2 (0, 40, 200 nM) in the presence or absence of the Caspase-1 inhibitor (Ac-YVAD-CHO). A strong 35 kDa BMPRIa fragment was observed at 200 nM BMP2, which was markedly reduced following Caspase-1 inhibition. β-actin (42 kDa) served as a loading control. * = *p* < 0.05, **** = *p* < 0.0001 and *n.s.* = not significant. (**B**) Densitometric quantification of the 35 kDa BMPRIa band intensity normalized to β-actin and expressed as fold change relative to control. Data represent mean ± SEM (*n* = 3; *p* < 0.05). Statistical analysis was performed using two-way ANOVA with Tukey’s post hoc test.

The cell lysates were collected and subjected to SDS-PAGE and Western blot analysis. Interestingly, the results revealed an additional BMPRIa fragment band with a molecular weight of about 35 kDa in the Caspase-1 uninhibited group, indicating cleavage (Figure 5). However, in the caspase-1 inhibited group, while the normal BMPRIa band was observed at 55 KDa, the cleavage band was absent or markedly reduced in intensity. Notably, BMPRIa appeared as multiple bands, which may be due to post-translational modification likely representing distinct glycosylated forms of the full-length receptor (~55 kDa) in addition to the ~35 kDa cleavage fragment. The disappearance of the 35 kDa band upon Caspase-1 inhibition supports the conclusion that this fragment arises from Caspase-1–mediated proteolysis rather than non-specific degradation. Moreover, because these are primary BMSCs isolated directly from mice, they may display post-translational modifications and cleavage patterns that differ from those observed in immortalized C2C12 cells, where receptor processing is less heterogeneous. These results show that Caspase-1 is indeed involved in the cleavage of the BMPRIa receptor with the inhibition of Caspase-1 abolishing the previously observed fragment.

Next, we tested the effects of Caspase-1 inhibition on BMPRIa localization to determine whether the nuclear translocation observed in the Caspase-1–uninhibited group would still occur under inhibited conditions.

### 3.6. Caspase-1 Inhibition Prevents the Nuclear Accumulation of BMPRIa in 6-Months-Old Primary BMSCs

**Figure 6.**
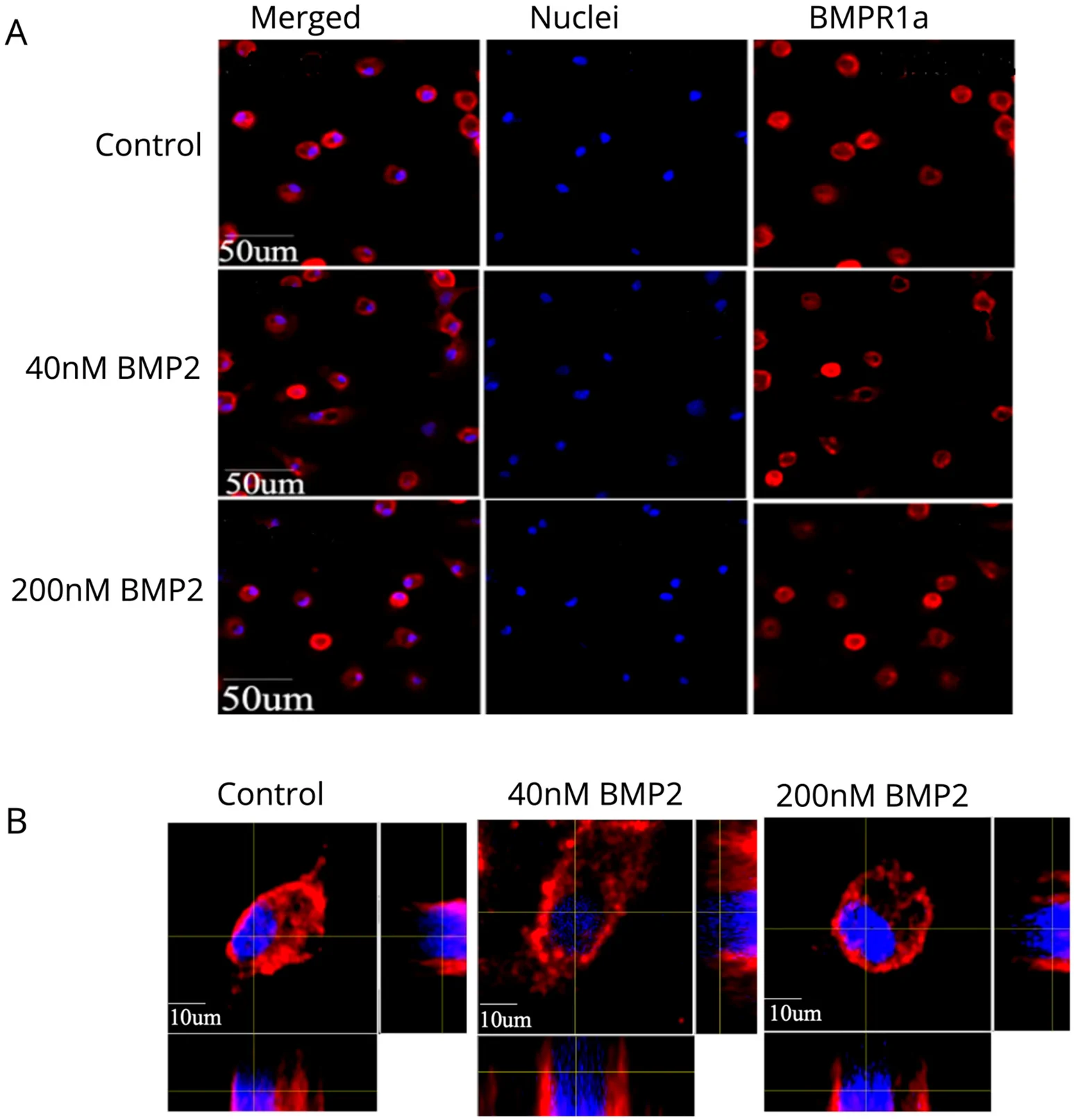
Effect of Caspase-1 inhibition on BMPRIa localization in BMSCs. (**A,B**) Immunofluorescence images of BMPRIa (red) in BMSCs pretreated with Caspase-1 inhibitor and stimulated with BMP2 (0, 40, 200 nM). Nuclei stained with Hoechst (blue).

Following the identification of Caspase-1 as the potential cleavage protein responsible for BMPRIa nuclear accumulation, we proceeded to test the effects of Caspase-1 inhibition and ascertain Caspase-1 involvement using a Caspase-1 inhibitor. Before BMP2 stimulation, the cells were treated with 12 μM of Caspase-1 inhibitor I (Ac-YVAD-CHO) for 24 h and then stimulated with BMP2 at 0 nM, 40 nM, and 200 nM as in previous experiments. Confocal microscopy was employed to obtain high-resolution images of the cells, which were subsequently analyzed using ImageJ software. The results revealed that inhibition of Caspase-1 prevented the nuclear accumulation of BMPRIa that was observed in the previous experiments without Caspase-1 inhibition. Here, in the presence of the Caspase-1 inhibitor, BMPRIa remained localized at the membrane and cytoplasmic region across all BMP2 treatment groups (Figure 6A,B).

These findings suggest that Caspase-1 is indeed involved in the cleavage of BMPRIa, leading to its nuclear translocation. The prevention of nuclear accumulation by Caspase-1 inhibition further implicates it in the regulation of BMP2 signaling pathways in primary BMSCs. This highlights the potential of targeting Caspase-1 to modulate BMPRIa localization and, consequently, the differentiation processes of BMSCs. Further, we investigated the effects of Caspase-1 inhibition on PPARγ expression.

### 3.7. Caspase-1 Inhibition Leads to a Reduction in PPARγ Expression in BMSC

To determine whether Caspase-1 activity contributes to BMP2-induced PPARγ expression, primary BMSCs were pre-treated with 12 μM Caspase-1 inhibitor I (Ac-YVAD-CHO) for 24 h prior to stimulation with BMP2 (0, 40, or 200 nM). Immunofluorescence staining and confocal imaging were performed to quantify PPARγ-positive cells and assess subcellular localization. Approximately 140 cells per group were analyzed across three independent experiments (*n* = 3; *p* < 0.05).

**Figure 7.**
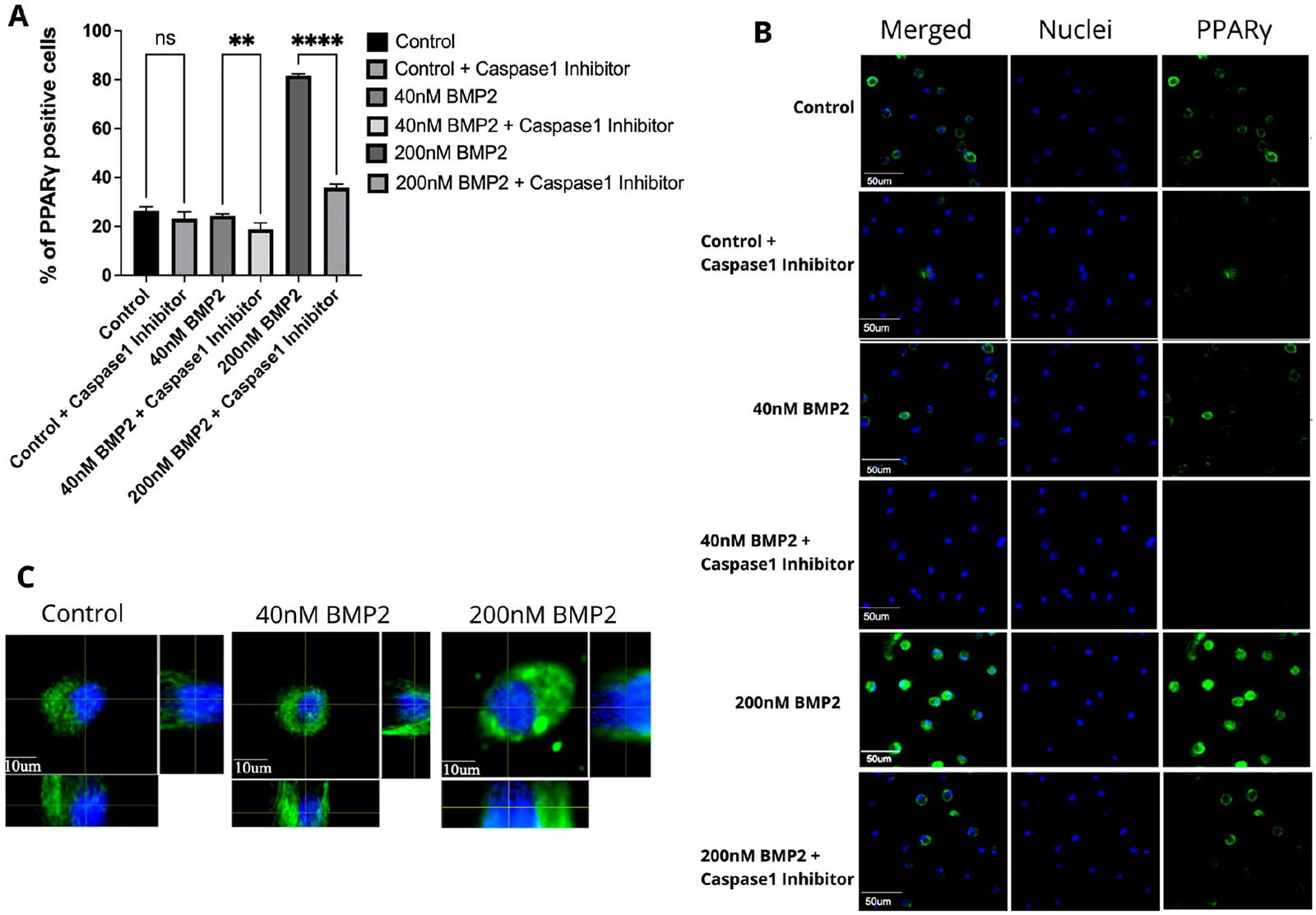
Effect of Caspase-1 inhibition on PPARγ expression in BMSCs. (**A**) Quantification of PPARγ-positive cells following BMP2 stimulation (0, 40, 200 nM) with or without Caspase-1 inhibitor (Ac-YVAD-CHO), ** = *p* < 0.01, **** = *p* < 0.0001 and *n.s.* = not significant. (**B**) Representative immunofluorescence images showing PPARγ expression (green) and nuclei (Hoechst, blue) in BMSCs under indicated conditions. Scale bars = 50 μm. (**C**) High-resolution z-stack orthogonal views illustrating subcellular distribution of PPARγ (green) in control, 40 nM, and 200 nM BMP2 groups. Scale bars = 10 μm. Quantitative analysis was performed across three independent experiments (*n* = 3; *p* < 0.05).

As shown in Figure 7A, Caspase-1 inhibition significantly reduced the percentage of PPARγ-positive cells across all BMP2-treated conditions, with the greatest reduction observed at 200 nM BMP2 (~55% decrease; *p* < 0.001) compared to the uninhibited group. Immunofluorescence images (Figure 7B) confirmed this trend. High-resolution z-stack images (Figure 7C) further demonstrated that upon Caspase-1 inhibition, PPARγ fluorescence shifted toward the cytoplasm in all the groups, including the 200 nM BMP2 condition, which previously exhibited strong nuclear localization in the uninhibited group. This redistribution indicates impaired activation and reduced nuclear translocation of PPARγ.

The observed reduction in PPARγ expression following Caspase-1 inhibition suggests that Caspase-1 activity is crucial for BMP2-mediated induction of PPARγ in BMSCs. Collectively, these findings demonstrate that Caspase-1 is essential for BMP2-induced upregulation and nuclear localization of PPARγ. Inhibition of Caspase-1 disrupts this signaling cascade, likely by preventing BMPRIa cleavage and subsequent nuclear signaling, thereby attenuating adipogenic differentiation.

### 3.8. High BMP2 Concentration Induces Adipogenesis and Caspase-1 Inhibition Leads to Its Reduction

To evaluate the combined effect of BMP2 stimulation and Caspase-1 inhibition on adipogenic differentiation of BMSCs, Oil Red O staining was performed to visualize intracellular lipid accumulation, a hallmark of mature adipocytes. Primary BMSCs were isolated and treated as described in the Materials and Methods section.

**Figure 8.**
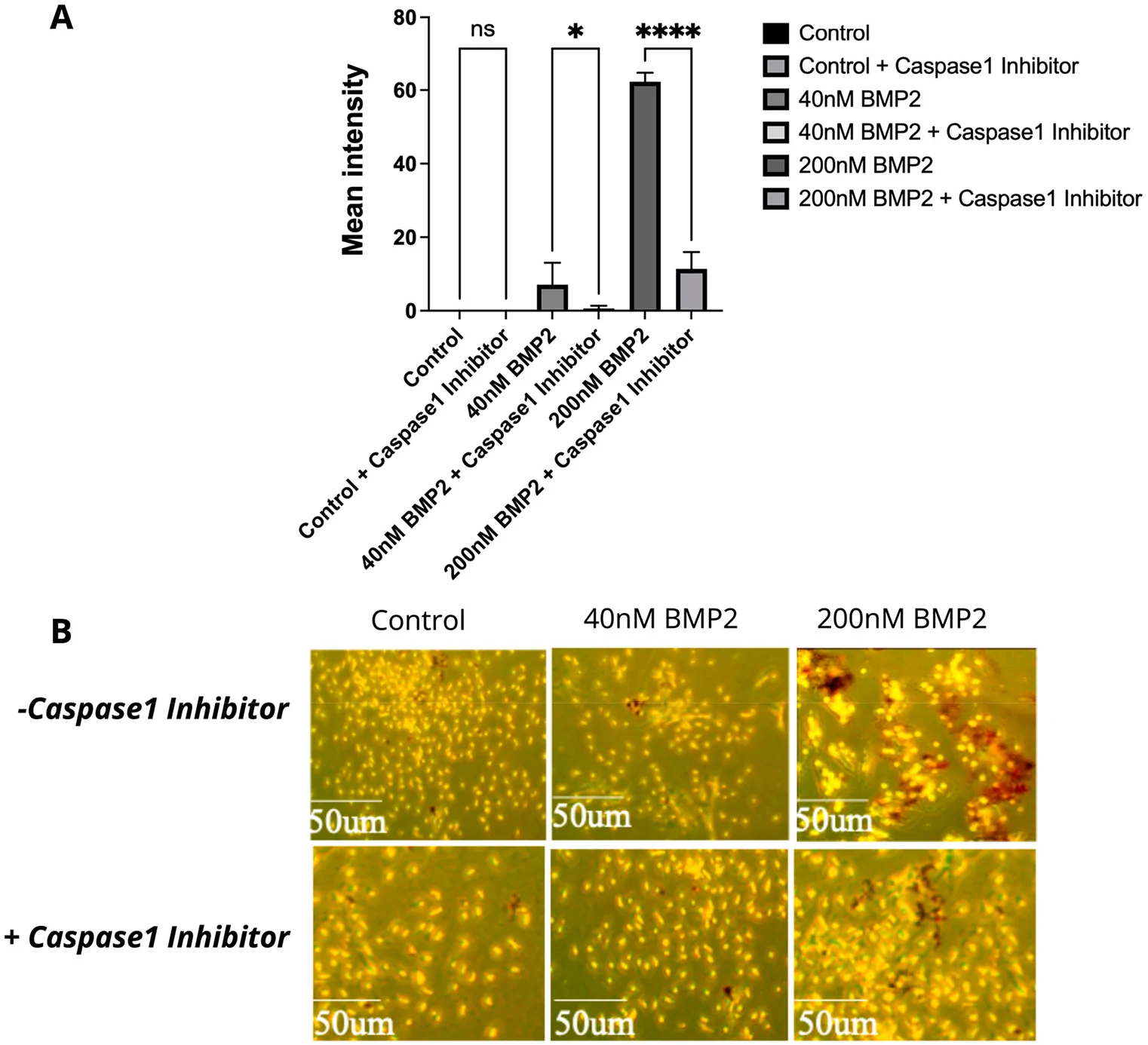
BMP2-induced adipogenesis and the effect of Caspase-1 inhibition in primary BMSCs. (**A**) Quantification of mean Oil Red O staining intensity in BMSCs treated with BMP2 (0, 40, 200 nM) in the presence or absence of Caspase-1 inhibitor (Ac-YVAD-CHO). * = *p* < 0.05, **** = *p* < 0.0001 and *n.s.* = not significant (**B**) Representative bright-field images showing lipid droplet accumulation (red staining) under the indicated conditions. Scale bars = 50 μm. Quantitative analysis was performed from three independent experiments (*n* = 3).

Cells treated with high BMP2 (200 nM) displayed a pronounced increase in lipid droplet formation, as evidenced by intense Oil Red O staining and higher mean intensity values compared to the control and 40 nM BMP2 groups (Figure 8A,B). Quantitative analysis showed an approximately 60% increase in lipid area coverage in the 200 nM BMP2 group, confirming that elevated BMP2 promotes adipogenesis in primary BMSCs.

In contrast, Caspase-1 inhibition markedly suppressed lipid accumulation across all treatment groups, with the greatest reduction observed at 200 nM BMP2. This reduction indicates that Caspase-1 activity is essential for BMP2-driven adipogenesis. These results are consistent with the immunofluorescence findings in Figure 7, which showed that Caspase-1 inhibition downregulated PPARγ, thereby limiting adipogenic commitment.

Collectively, these results demonstrate that BMP2 promotes adipogenesis in a concentration-dependent manner, while Caspase-1 inhibition significantly impairs lipid accumulation and adipogenic differentiation in primary BMSCs.

## 4. Discussion

As the global population continues to age, the prevalence of bone disorders such as osteoporosis is increasing, creating an urgent need for effective therapeutic approaches [53,54]. These conditions are characterized by an imbalance between bone formation and resorption, a process exacerbated by the age-related decline in bone marrow mesenchymal stem cell (BMSC) function [55,56]. Bone Morphogenetic Protein 2 (BMP2) has emerged as a key regulator of BMSC fate due to its ability to promote both osteogenic and adipogenic differentiation, depending on concentration and cellular context [5]. While lower BMP2 concentrations have been associated with osteogenesis, higher concentrations can trigger adipogenic differentiation, thereby complicating its therapeutic use [47].

In this study, we provide evidence that Caspase-1 mediates the proteolytic cleavage of BMPRIa in response to high BMP2 concentrations in BMSCs, and that this cleavage is associated with nuclear translocation of BMPRIa and increased expression of PPARγ, a master regulator of adipogenesis. These findings extend previous work by [42], who first described BMP2-induced BMPRIa cleavage, and provide new mechanistic insight into how BMP2 signaling promotes adipogenesis. Specifically, our Western blot analysis demonstrated BMP2-induced cleavage in both C2C12 and primary BMSCs, with a prominent cleavage band appearing at 200 nM BMP2 (Figure 1). This aligns with prior work by [42] but extends those findings by identifying Caspase-1 as the likely protease responsible for BMPRIa cleavage.

Functionally, we observed that high BMP2 concentrations significantly increased PPARγ expression in BMSCs, as shown by immunofluorescence staining and quantification (Figure 2). Since PPARγ is a central regulator of adipogenesis, this increase is consistent with BMP2-induced adipogenic signaling [8,57]. At the same time, cells stimulated with 40 nM BMP2 exhibited fewer PPARγ-positive nuclei, consistent with osteogenic differentiation as reported by [47]. Confocal imaging further revealed altered BMPRIa localization depending on BMP2 concentration. At 200 nM BMP2, BMPRIa accumulated in the nucleus (Figure 3), whereas at 40 nM BMP2 it remained predominantly on the membrane or cytoplasm. This suggests that cleavage and nuclear trafficking of BMPRIa are concentration-dependent events that may underlie the switch between osteogenesis and adipogenesis. A predicted Caspase-1 cleavage site at amino acid 310 of BMPRIa supports the plausibility of Caspase-1–dependent processing (Figure 4).

Indeed, Caspase-1 inhibition markedly reduced BMPRIa cleavage in BMP2-treated BMSCs (Figure 5), prevented its nuclear accumulation (Figure 6), and suppressed PPARγ expression (Figure 7). These results strongly implicate Caspase-1 in regulating BMPRIa cleavage and downstream adipogenic signaling. Furthermore, Oil Red O staining demonstrated increased lipid accumulation in BMSCs treated with high BMP2, which was significantly reduced upon Caspase-1 inhibition (Figure 8). Together, these data suggest that Caspase-1 activity is essential for BMP2-induced BMPRIa cleavage, nuclear translocation, and subsequent PPARγ-driven adipogenic differentiation in BMSCs.

Our findings expand on prior work in several ways. First, while certain BMP family members have been shown to undergo metalloproteinase-mediated cleavage (e.g., activation of BMP-7 by MMP-13) [58], our data identify a novel role for Caspase-1 as a regulatory protease within the BMP2 signaling pathway. Caspase-1 is best known for its role in pyroptosis and cytokine maturation [59]; however, our results suggest a noncanonical role in lineage specification, linking BMP2 signaling to metabolic differentiation pathways. Understanding how Caspase-1 biases BMP2 signaling toward adipogenesis is therefore highly relevant for therapeutic applications.

Furthermore, the inability of BMPRIa to translocate to the nucleus under Caspase-1 inhibition suggests that the cleavage fragment generated by Caspase-1 may act as a nuclear effector that modulates gene transcription. This provides a novel insight into how proteolytic regulation of BMP receptors could fine-tune downstream gene expression and offers a potential strategy for modulating BMP2 signaling outcomes by targeting Caspase-1 activity. Similar cleavage-dependent nuclear signaling mechanisms have been described for receptors such as Notch and EGFR, supporting the biological plausibility of BMPRIa functioning as a nuclear effector after Caspase-1–mediated cleavage [60].

**Figure 9.**
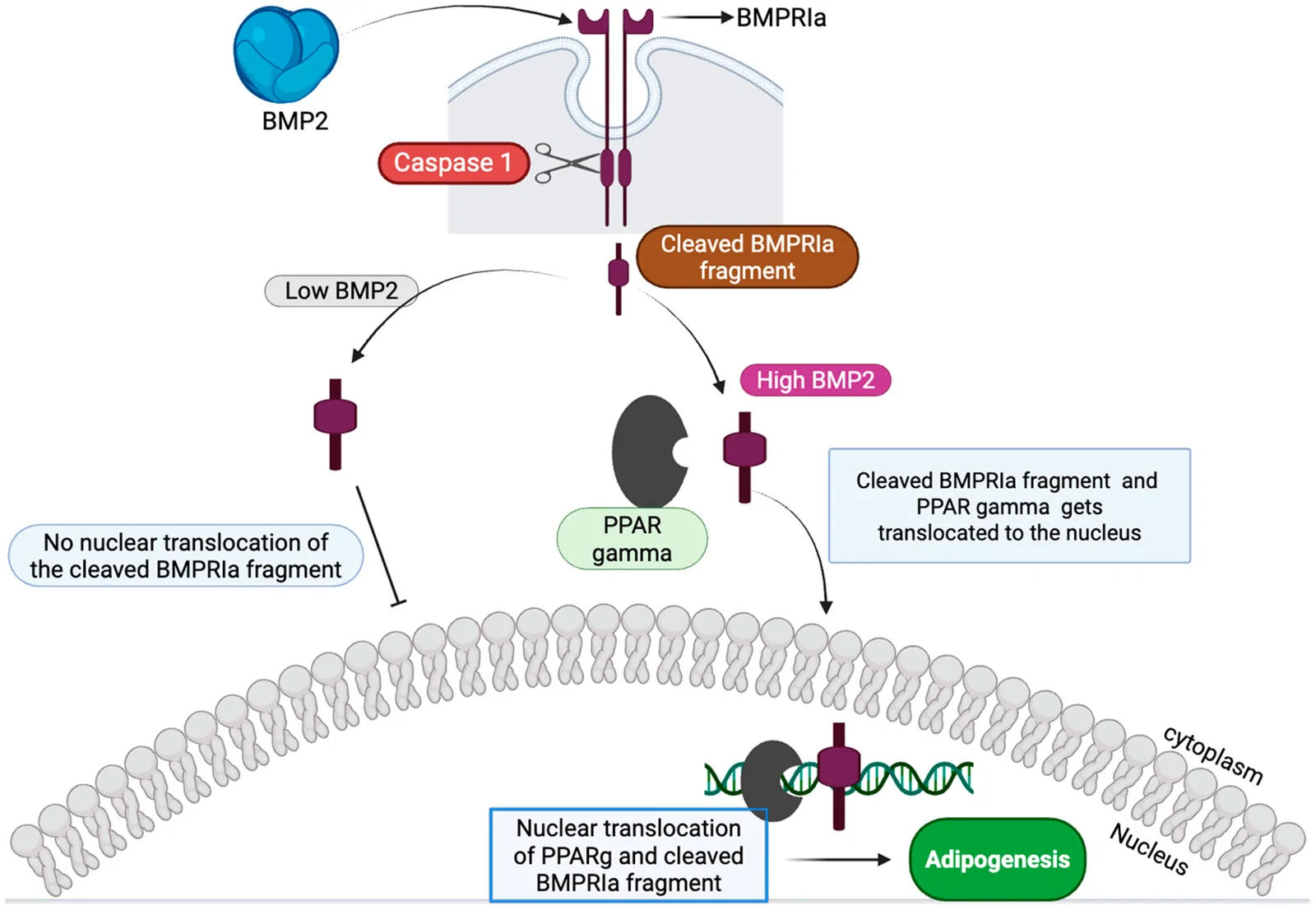
Putative model of Caspase-1–mediated BMPRIa cleavage and nuclear translocation. High BMP2 concentration induces Caspase-1 cleavage of BMPRIa, generating a fragment that translocates to the nucleus together with PPARγ to promote adipogenesis. At lower BMP2 concentration, BMPRIa remains membrane-associated, PPARγ nuclear translocation is limited, and osteogenesis is favored.

Based on these results, we propose a model in which high BMP2 concentrations activate Caspase-1–mediated cleavage of BMPRIa, generating a fragment that translocates to the nucleus, where it cooperates with or enhances PPARγ transcriptional activity to drive adipogenic signaling. Conversely, at lower BMP2 concentrations, BMPRIa cleavage is reduced, nuclear translocation is limited, and osteogenesis is favored. This model is summarized in Figure 9.

It is also important to consider the translational relevance of these findings. Although our experiments were conducted using murine BMSCs, sequence alignment of murine and human BMPRIa revealed more than 97% amino acid identity, with conservation of the predicted Caspase-1 cleavage site at position 310. This high degree of homology supports the applicability of our findings to human systems, suggesting that Caspase-1–mediated receptor cleavage is likely a conserved regulatory mechanism.

Understanding the molecular mechanisms underlying BMPRIa cleavage and its regulation by Caspase-1 could inform the development of novel therapeutic strategies aimed at enhancing BMP2 signaling in osteoporotic patients. By specifically targeting the proteolytic processing pathways, it may be possible to restore the osteogenic potential of BMSCs and mitigate the progression of age-related bone loss. However, while Caspase-1 has been identified as the primary BMPRIa protease, other proteases may also contribute to this cleavage. Future studies should explore whether additional proteolytic or post-translational modifications influence BMPRIa’s nuclear function and downstream differentiation outcomes.

In summary, high BMP2 concentrations (200 nM) induce Caspase-1–mediated cleavage of BMPRIa, facilitating its nuclear translocation and interaction with PPARγ, thereby promoting adipogenic differentiation in BMSCs. At lower BMP2 concentrations (40 nM), BMPRIa remains membrane-associated and fails to activate nuclear PPARγ signaling, favoring osteogenic over adipogenic differentiation.

### Limitations and Future Directions

While this study provides valuable insights into BMP2-induced signaling in BMSCs, several limitations must be acknowledged. The use of primary BMSCs from mice may not fully recapitulate the human disease context, and thus, further validation in human cells and clinical samples is warranted. Additionally, the specific molecular mechanisms by which BMPRIa nuclear fragments regulate gene transcription remain to be elucidated, necessitating further investigation into the potential transcriptional targets and binding partners of these fragments, especially in human cells.

We also did not directly demonstrate Caspase-1 binding to BMPRIa or co-immunoprecipitate the cleavage fragment. Time-course experiments would be valuable to confirm whether cleavage precedes PPARγ nuclear translocation. In addition, our primary BMSCs were not characterized by flow cytometry for stemness markers, and adipogenesis was assessed primarily by PPARγ expression and Oil Red O staining, rather than by additional markers such as Adipoq or FABP4. These limitations suggest that our findings reflect adipogenic potential rather than fully established adipocyte differentiation.

Furthermore, the BMP2 concentrations used here are supraphysiological but were selected based on established in vitro models that distinguish osteogenic and adipogenic outcomes. Future work should test whether similar mechanisms operate at lower, physiologically relevant doses and in human BMSCs.

Finally, while Caspase-1 was identified as the primary protease mediating BMPRIa cleavage, other proteases may also contribute. Determining whether metalloproteinases or additional caspases act in parallel will be important. Translationally, our findings suggest that targeting Caspase-1 activity could modulate BMP2 signaling outcomes, enhancing osteogenesis while limiting adipogenesis in the bone marrow niche, a strategy that could hold promise for osteoporosis and other age-related bone disorders.

## Data Availability

Any data or material that support the findings of this study can be made available by the corresponding author upon request.
